# New double inequalities for g-frames in Hilbert $C^{*}$-modules

**DOI:** 10.1186/s40064-016-2731-2

**Published:** 2016-07-08

**Authors:** Zhong-Qi Xiang

**Affiliations:** College of Mathematics and Computer Science, Shangrao Normal University, Shangrao, 334001 People’s Republic of China

**Keywords:** Hilbert $C^{*}$-module, g-Frame, Inequality, Scalar, 46L99, 42C15, 46H25

## Abstract

In this work, we establish two types of double inequalities for g-frames in Hilbert $C^{*}$-modules, which involve scalars $\lambda \in [0,1]$ and $\lambda \in [\frac{1}{2},1]$ respectively. It is shown that the results we obtained can immediately lead to the existing corresponding results when taking $\lambda =\frac{1}{2}$.

## Background

The origins of the notion of frames can be traced back to the literature Duffin and Schaeffer (1952) in the early 1950’s, when they were used to deal with some problems in nonharmonic Fourier series. People did not realize the importance of frames until the publication of the fundamental paper Daubechies et al. (1986) on wavelet theory due to Daubechies, Grossmann and Meyer. Today, owing to the flexibility, frames have been used in dozens of areas by applied mathematicians and engineers (see Benedetto et al. 2006; Candès and Donoho 2005; Sun (2010). Sun (2006) proposed the concept of g-frames which extends the concept of frames from bounded linear functionals to operators and covers many recent generalizations of frames.

On the other hand, the concepts of frames and g-frames for Hilbert spaces have been generalized to the case of Hilbert $C^{*}$-modules (Frank and Larson 2002; Khosravi and Khosravi 2008). It should be pointed out, due to the complex structure of $C^{*}$-algebras embedded in the Hilbert $C^{*}$-modules, that the problems about frames and g-frames in Hilbert $C^{*}$-modules are more complicated than those in Hilbert spaces. Frames and especial g-frames for Hilbert $C^{*}$-modules have been studied intensively, for more details see Alijani (2015), Alijani and Dehghan (2012), Askarizadeh and Dehghan (2013), Han et al. (2013), Rashidi-Kouchi et al. (2014), Xiang and Li (2016), Xiang (2016), Xiao and Zeng (2010).

We need to collect some notations and basic definitions.

Throughout this paper, the symbols ${\mathbb {J}}$ and ${\mathcal {A}}$ are reserved for a finite or countable index set and a unital $C^{*}$-algebra, respectively. ${\mathscr {H}}$, ${\mathscr {K}}$ and ${\mathscr {K}}_{j}$’s are Hilbert $C^{*}$-modules over ${\mathcal {A}}$, and put $\langle f,f\rangle =|f|^{2}$ for every $f\in \mathscr {H}$. We use $\mathrm{End}^{*}_{\mathcal {A}}(\mathscr {H},\mathscr {K})$ to denote the set of all adjointable operators from ${\mathscr {H}}$ to ${\mathscr {K}}$, and $\mathrm{End}^{*}_{\mathcal {A}}(\mathscr {H},\mathscr {H})$ is abbreviated to $\mathrm{End}^{*}_{\mathcal {A}}(\mathscr {H})$.

A family $\{\Lambda _{j}\in \mathrm{End}^{*}_{\mathcal {A}} ({\mathscr {H}},{\mathscr {K}}_{j})\}_{j\in \mathbb {J}}$ is said to be a g-frame for ${\mathscr {H}}$ with respect to $\{{\mathscr {K}}_{j}\}_{j\in \mathbb {J}}$, if there are two positive constants $0<C\le D<\infty$ such that

1 $$C\langle f,f\rangle \le \sum _{j\in \mathbb {J}}\langle \Lambda _{j}f,\Lambda _{j}f\rangle \le D\langle f,f\rangle ,\quad \forall \,f\in \mathscr {H}.$$

The numbers *C* and *D* are called g-frame bounds. We call $\{\Lambda _{j}\}_{j\in {\mathbb {J}}}$ a $\lambda$-tight g-frame if $C=D=\lambda$, and a Parseval g-frame if $C=D=1$. The sequence $\{\Lambda _{j}\}_{j\in \mathbb {J}}$ is called a g-Bessel sequence with bound *D* if only the right hand inequality of (1) is satisfied.

Let $\{\Lambda _{j}\in \mathrm{End}^{*}_{\mathcal {A}} ({\mathscr {H}},{\mathscr {K}}_{j})\}_{j\in \mathbb {J}}$ be a g-frame for ${\mathscr {H}}$ with respect to $\{{\mathscr {K}}_{j}\}_{j\in \mathbb {J}}$, then the g-frame operator *S* for $\{\Lambda _{j}\}_{j\in \mathbb {J}}$ is defined by

2 $$S:{\mathscr {H}}\rightarrow {\mathscr {H}},\quad Sf=\sum _{j\in \mathbb {J}}\Lambda _{j}^{*}\Lambda _{j}f,\quad \forall \,f\in \mathscr {H}.$$

It is easily seen that *S* is positive, self-adjoint and invertible. Denote $\widetilde{\Lambda }_{j}=\Lambda _{j}S^{-1}$ for each $j\in \mathbb {J}$, then a simple calculation shows that $\{\widetilde{\Lambda }_{j}\}_{j\in \mathbb {J}}$ remains a g-frame for ${\mathscr {H}}$ with respect to $\{{\mathscr {K}}_{j}\}_{j\in \mathbb {J}}$, which we call the canonical dual g-frame of $\{\Lambda _{j}\}_{j\in \mathbb {J}}$. For any ${\mathbb {K}}\subset {\mathbb {J}}$, we let ${\mathbb {K}}^{c}=\mathbb {J}\backslash \mathbb {K}$, and define the adjointable operators

3 $$S_{\mathbb {K}},S_{{\mathbb {K}}^{c}}:\mathscr {H}\rightarrow \mathscr {H},\quad S_{\mathbb {K}}f=\sum _{j\in \mathbb {K}}\Lambda ^{*}_{j}\Lambda _{j}f,\quad S_{{\mathbb {K}}^{c}}f=\sum _{j\in {\mathbb {K}}^{c}}\Lambda ^{*}_{j}\Lambda _{j}f,\quad \forall \,f\in \mathscr {H}.$$

Balan et al. (2007) discovered a remarkable inequality for Parseval frames in Hilbert spaces when working on efficient algorithms for signal reconstruction. Later on, Găvruţa (2006) extended it to general frames. The results of Găvruţa (2006) were applied recently in quantum information theory, see Jivulescu and Găvruţa (2015). Moreover, Poria Poria (2016) generalized those inequalities to the case of Hilbert–Schmidt frames, which possess a more general form. On the other hand, the authors of Xiao and Zeng (2010) have already extended the inequalities for Parseval frames and general frames to g-frames in Hilbert $C^{*}$-modules:

### **Theorem 1**

*Let *$\{\Lambda _{j}\in \mathrm{End}^{*}_{\mathcal {A}} ({\mathscr {H}},{\mathscr {K}}_{j})\}_{j\in \mathbb {J}}$*be a g-frame for*${\mathscr {H}}$*with respect to*$\{{\mathscr {K}}_{j}\}_{j\in \mathbb {J}}$, *and*$\{\widetilde{\Lambda }_{j}\} _{j\in \mathbb {J}}$*be the canonical dual g-frame of*$\{\Lambda _{j}\}_{j\in \mathbb {J}}$, *then for any*${\mathbb {K}}\subset {\mathbb {J}}$*and any*$f\in \mathscr {H}$, *we have*

4 $$\sum _{j\in \mathbb {J}}|\widetilde{\Lambda }_{j}S_{\mathbb {K}}f|^{2}+\sum _{j\in {\mathbb {K}}^{c}}|\Lambda _{j}f|^{2}= \sum _{j\in \mathbb {J}}|\widetilde{\Lambda }_{j}S_{{\mathbb {K}}^{c}}f|^{2}+\sum _{j\in \mathbb {K}}|\Lambda _{j}f|^{2} \ge \frac{3}{4}\sum _{j\in \mathbb {J}}|\Lambda _{j}f|^{2}.$$

### **Theorem 2**

*Let *$\{\Lambda _{j}\in \mathrm{End}^{*}_{\mathcal {A}} ({\mathscr {H}},{\mathscr {K}}_{j})\}_{j\in \mathbb {J}}$*be a Parseval g-frame for*${\mathscr {H}}$*with respect to*$\{{\mathscr {K}}_{j}\}_{j\in \mathbb {J}}$, *then for any*${\mathbb {K}}\subset {\mathbb {J}}$*and any*$f\in \mathscr {H}$, *we have*

5 $$\biggl |\sum _{j\in \mathbb {K}}\Lambda ^{*}_{j}\Lambda _{j}f\biggl |^{2}+\sum _{j\in {\mathbb {K}}^{c}}|\Lambda _{j}f|^{2}= \biggl |\sum _{j\in {\mathbb {K}}^{c}}\Lambda ^{*}_{j}\Lambda _{j}f\biggl |^{2}+\sum _{j\in \mathbb {K}}|\Lambda _{j}f|^{2}\ge \frac{3}{4}\langle f,f\rangle.$$

Recently, the author of Xiang (2016) obtained several new inequalities for g-frames in Hilbert $C^{*}$-modules which are different in structure from (4) and (5):

### **Theorem 3**

*Let*$\{\Lambda _{j}\in \mathrm{End}^{*}_{\mathcal {A}}({\mathscr {H}},{\mathscr {K}}_{j})\}_{j\in \mathbb {J}}$*be a g-frame for*${\mathscr {H}}$*with respect to*$\{{\mathscr {K}}_{j}\}_{j\in \mathbb {J}}$*with canonical dual g-frame *$\{\widetilde{\Lambda }_{j}\} _{j\in \mathbb {J}}$. *Then for any*$\mathbb {K}\subset \mathbb {J}$*and any*$f\in \mathscr {H}$, *we have*

6 $$\begin{aligned}&0\le \sum _{j\in \mathbb {K}}|\Lambda _{j}f|^{2}-\sum _{j\in \mathbb {J}}|\widetilde{\Lambda }_{j}S_{\mathbb {K}}f|^{2}\le \frac{1}{4}\sum _{j\in \mathbb {J}}|\Lambda _{j}f|^{2}. \end{aligned}$$

7 $$\begin{aligned}&\frac{1}{2}\sum _{j\in \mathbb {J}}|\Lambda _{j}f|^{2}\le \sum _{j\in \mathbb {J}}|\widetilde{\Lambda }_{j}S_{\mathbb {K}}f|^{2}+\sum _{j\in \mathbb {J}}|\widetilde{\Lambda }_{j}S_{{\mathbb {K}}^{c}}f|^{2}\le \sum _{j\in \mathbb {J}}|\Lambda _{j}f|^{2}. \end{aligned}$$

### **Theorem 4**

*Let*$\{\Lambda _{j}\in \mathrm{End}^{*}_{\mathcal {A}} ({\mathscr {H}},{\mathscr {K}}_{j})\}_{j\in \mathbb {J}}$*be a Parseval g-frame for*${\mathscr {H}}$*with respect to*$\{{\mathscr {K}}_{j}\}_{j\in \mathbb {J}}$. *Then for any*${\mathbb {K}}\subset {\mathbb {J}}$*and any*$f\in \mathscr {H}$, *we have*

8 $$\begin{aligned}&0\le \sum _{j\in \mathbb {K}}|\Lambda _{j}f|^{2}-\biggl |\sum _{j\in \mathbb {K}}\Lambda ^{*}_{j}\Lambda _{j}f\biggl |^{2}\le \frac{1}{4}\langle f,f\rangle . \end{aligned}$$

9 $$\begin{aligned}&\frac{1}{2}\langle f,f\rangle \le \biggl |\sum _{j\in \mathbb {K}}\Lambda ^{*}_{j}\Lambda _{j}f\biggl |^{2}\,+\,\biggl |\sum _{j\in {\mathbb {K}}^{c}}\Lambda ^{*}_{j}\Lambda _{j}f\biggl |^{2}\le \langle f,f\rangle . \end{aligned}$$

Inspired by the idea of Poria (2016), in this paper we present two types of double inequalities for g-frames in Hilbert $C^{*}$-modules where the scalars $\lambda \in [0,1]$ and $\lambda \in [\frac{1}{2},1]$ are involved respectively, and we show that inequalities (4–9) can be obtained for a special value of $\lambda =\frac{1}{2}$.

## The main results and their proofs

To derive our main results, we need the following simple result for operators. It should be remarked that the fist part of this result is a generalization of Proposition 3.4 in Poria (2016). Although the proof is based on modification of the proof in Poria (2016), we include the proof for the sake of completeness.

### **Lemma 5**

*If*$U,V\in \mathrm{End}^{*}_{\mathcal {A}}(\mathscr {H})$*are self-adjoint operators satisfying*$U+V=\mathrm{Id}_{\mathscr {H}}$, *then for any*$\lambda \in [0,1]$*and any*$f\in \mathscr {H}$*we have*

10 $$\begin{aligned} \langle Uf,Uf\rangle +2\lambda \langle Vf,f\rangle&=\langle Vf,Vf\rangle +2(1-\lambda )\langle Uf,f\rangle \nonumber \\&\qquad +(2\lambda -1)\langle f,f\rangle \nonumber \\&\ge (2\lambda -\lambda ^{2})\langle f,f\rangle . \end{aligned}$$

* Moreover, if U and V are positive, then for any*$\lambda \in [\frac{1}{2},1]$*and any*$f\in \mathscr {H}$*we have*

11 $$\begin{aligned} \langle Uf,Uf\rangle \le 2\lambda \langle Uf,f\rangle ,\quad \langle Vf,Vf\rangle \le 2\lambda \langle Vf,f\rangle . \end{aligned}$$

### *Proof*

For any $\lambda \in [0,1]$ and any $f\in \mathscr {H}$, we have

12 $$\begin{aligned}&\langle Uf,Uf\rangle +2\lambda \langle Vf,f\rangle \nonumber \\&\qquad =\langle U^{2}f,f\rangle +2\lambda \langle (\mathrm{Id}_{\mathscr {H}}-U)f,f\rangle \nonumber \\&\qquad =\langle (U^{2}-2\lambda U+2\lambda \mathrm{Id}_{\mathscr {H}})f,f\rangle \nonumber \\&\qquad =\langle (\mathrm{Id}_{\mathscr {H}}-U)^{2}f,f\rangle +2(1-\lambda )\langle Uf,f\rangle +(2\lambda -1)\langle f,f\rangle \nonumber \\&\qquad =\langle Vf,Vf\rangle +2(1-\lambda )\langle Uf,f\rangle +(2\lambda -1)\langle f,f\rangle . \end{aligned}$$

We also have

13 $$\begin{aligned} \langle (U^{2}-2\lambda U+2\lambda \mathrm{Id}_{\mathscr {H}})f,f\rangle&=\langle ((U-\lambda \mathrm{Id}_{\mathscr {H}})^{2}- \lambda ^{2}\mathrm{Id}_{\mathscr {H}}+2\lambda \mathrm{Id}_{\mathscr {H}})f,f\rangle \nonumber \\&=\langle (U-\lambda \mathrm{Id}_{\mathscr {H}})^{2}f,f\rangle +\langle (2\lambda -\lambda ^{2})f,f\rangle \nonumber \\&=\langle (U-\lambda \mathrm{Id}_{\mathscr {H}})f,(U-\lambda \mathrm{Id}_{\mathscr {H}})f\rangle +(2\lambda -\lambda ^{2})\langle f,f\rangle \nonumber \\&\ge (2\lambda -\lambda ^{2})\langle f,f\rangle . \end{aligned}$$

This along with (12) leads to (10). We next prove that (11) holds. Since *U* and *V* are positive operators and $UV=VU$, we obtain

$$\begin{aligned} 0\le UV=U(\mathrm{Id}_{\mathscr {H}}-U)=U-U^{2}. \end{aligned}$$

Then, for any $\lambda \in [\frac{1}{2},1]$ and any $f\in \mathscr {H}$ we get

$$\begin{aligned} \langle Uf,Uf\rangle +2\lambda \langle Vf,f\rangle&\le \langle Uf,f\rangle +2\lambda \langle (\mathrm{Id}_{\mathscr {H}}-U)f,f\rangle \\&=(1-2\lambda )\langle Uf,f\rangle +2\lambda \langle f,f\rangle \le 2\lambda \langle f,f\rangle , \end{aligned}$$

it follows that

$$\begin{aligned} \langle Uf,Uf\rangle \le 2\lambda \langle f,f\rangle -2\lambda \langle Vf,f\rangle =2\lambda \langle Uf,f\rangle . \end{aligned}$$

The second inequality in (11) can be proved similarly.

### **Theorem 6**

*Let*$\{\Lambda _{j}\in \mathrm{End}^{*}_{\mathcal {A}}({\mathscr {H}},{\mathscr {K}}_{j})\}_{j\in \mathbb {J}}$*be a g-frame for*$\mathscr {H}$*with respect to*$\{{\mathscr {K}}_{j}\}_{j\in \mathbb {J}}$, *and*$\{\widetilde{\Lambda }_{j}\}$*be the canonical dual g-frame of*$\{\Lambda _{j}\}_{j\in \mathbb {J}}$, *then for any*$\lambda \in [0,1]$, *for all*$\mathbb {K}\subset \mathbb {J}$*and all*$f\in \mathscr {H}$, *we have*

14 $$\begin{aligned} \sum _{j\in \mathbb {J}}|\Lambda _{j}f|^{2}&\ge \sum _{j\in \mathbb {J}}|\widetilde{\Lambda }_{j} S_{\mathbb {K}}f|^{2}+\sum _{j\in {\mathbb {K}}^{c}}|\Lambda _{j}f|^{2} =\sum _{j\in \mathbb {J}}|\widetilde{\Lambda }_{j}S_{{\mathbb {K}}^{c}}f|^{2}+\sum _{j\in \mathbb {K}}|\Lambda _{j}f|^{2}\nonumber \\&\ge (2\lambda -\lambda ^{2})\sum _{j\in \mathbb {K}}|\Lambda _{j}f|^{2}+(1-\lambda ^{2})\sum _{j\in {\mathbb {K}}^{c}}|\Lambda _{j}f|^{2}. \end{aligned}$$

### *Proof*

Denote by *S* the g-frame operator of $\{\Lambda _{j}\}_{j\in \mathbb {J}}$, then $S_{\mathbb {K}}+S_{{\mathbb {K}}^{c}}=S$ and, $S^{-\frac{1}{2}}S_{\mathbb {K}}S^{-\frac{1}{2}} +S^{-\frac{1}{2}}S_{{\mathbb {K}}^{c}}S^{-\frac{1}{2}}=\mathrm{Id}_{\mathscr {H}}$ as a consequence. Taking $S^{-\frac{1}{2}}S_{\mathbb {K}}S^{-\frac{1}{2}}$, $S^{-\frac{1}{2}}S_{{\mathbb {K}}^{c}}S^{-\frac{1}{2}}$ and $S^{\frac{1}{2}}f$ instead of *U*, *V* and *f* respectively in Lemma 5 yields

$$\begin{aligned}&\langle S^{-1}S_{{\mathbb {K}}^{c}}f,S_{{\mathbb {K}}^{c}}f\rangle +2(1-\lambda )\langle S_{\mathbb {K}}f,f\rangle +(2\lambda -1)\langle Sf,f\rangle \\&\qquad \ge (2\lambda -\lambda ^{2})\langle S^{\frac{1}{2}}f,S^{\frac{1}{2}}f\rangle =(2\lambda -\lambda ^{2})\langle Sf,f\rangle . \end{aligned}$$

It follows that

15 $$\begin{aligned} \langle S^{-1}S_{\mathbb {K}}f,S_{\mathbb {K}}f\rangle &=\langle S^{-1}S_{{\mathbb {K}}^{c}}f,S_{{\mathbb {K}}^{c}}f\rangle +2(1-\lambda )\langle S_{\mathbb {K}}f,f\rangle \nonumber \\&\qquad +(2\lambda -1)\langle Sf,f\rangle -2\lambda \langle S_{{\mathbb {K}}^{c}}f,f\rangle \nonumber \\&\ge (2\lambda -\lambda ^{2})\langle Sf,f\rangle -2\lambda \langle S_{{\mathbb {K}}^{c}}f,f\rangle \nonumber \\&=2\lambda (\langle Sf,f\rangle -\langle S_{{\mathbb {K}}^{c}}f,f\rangle )-\lambda ^{2}\langle Sf,f\rangle \nonumber \\&=2\lambda \langle S_{\mathbb {K}}f,f\rangle -\lambda ^{2}\langle Sf,f\rangle . \end{aligned}$$

Noting that

16 $$\begin{aligned}&\langle S^{-1}S_{\mathbb {K}}f,S_{\mathbb {K}}f\rangle +2\lambda \langle S_{{\mathbb {K}}^{c}}f,f\rangle \nonumber \\&\qquad =\langle S^{-1}S_{{\mathbb {K}}^{c}}f,S_{{\mathbb {K}}^{c}}f\rangle +2(1-\lambda )\langle S_{\mathbb {K}}f,f\rangle +(2\lambda -1)\langle Sf,f\rangle \nonumber \\&\qquad =\langle S^{-1}S_{{\mathbb {K}}^{c}}f,S_{{\mathbb {K}}^{c}}f\rangle +2\langle S_{\mathbb {K}}f,f\rangle +2\lambda (\langle Sf,f\rangle -\langle S_{\mathbb {K}}f,f\rangle )-\langle Sf,f\rangle \nonumber \\&\qquad =\langle S^{-1}S_{{\mathbb {K}}^{c}}f,S_{{\mathbb {K}}^{c}}f\rangle +\langle S_{\mathbb {K}}f,f\rangle -\langle S_{{\mathbb {K}}^{c}}f,f\rangle +2\lambda \langle S_{{\mathbb {K}}^{c}}f,f\rangle , \end{aligned}$$

we have

17 $$\begin{aligned} \langle S^{-1}S_{{\mathbb {K}}^{c}}f,S_{{\mathbb {K}}^{c}}f\rangle +\langle S_{\mathbb {K}}f,f\rangle&=\langle S^{-1}S_{\mathbb {K}}f,S_{\mathbb {K}}f\rangle +\langle S_{{\mathbb {K}}^{c}}f,f\rangle \nonumber \\&\ge 2\lambda \langle S_{\mathbb {K}}f,f\rangle -\lambda ^{2}\langle Sf,f\rangle +\langle S_{{\mathbb {K}}^{c}}f,f\rangle \nonumber \\&=(2\lambda -\lambda ^{2})\langle S_{\mathbb {K}}f,f\rangle +(1-\lambda ^{2})\langle S_{{\mathbb {K}}^{c}}f,f\rangle . \end{aligned}$$

For each $f\in \mathscr {H}$, since

18 $$\begin{aligned} \sum _{j\in \mathbb {J}}|\widetilde{\Lambda }_{j}S_{\mathbb {K}}f|^{2}+\sum _{j\in {\mathbb {K}}^{c}}|\Lambda _{j}f|^{2}&=\langle SS^{-1}S_{\mathbb {K}}f,S^{-1}S_{\mathbb {K}}f\rangle +\langle S_{{\mathbb {K}}^{c}}f,f\rangle \nonumber \\&=\langle S^{-1}S_{\mathbb {K}}f,S_{\mathbb {K}}f\rangle +\langle S_{{\mathbb {K}}^{c}}f,f\rangle \nonumber \\&=\langle S^{-1}S_{{\mathbb {K}}^{c}}f,S_{{\mathbb {K}}^{c}}f\rangle +\langle S_{\mathbb {K}}f,f\rangle \nonumber \\&=\langle SS^{-1}S_{{\mathbb {K}}^{c}}f,S^{-1}S_{{\mathbb {K}}^{c}}f\rangle +\langle S_{\mathbb {K}}f,f\rangle \nonumber \\&=\sum _{j\in \mathbb {J}}|\widetilde{\Lambda }_{j}S_{{\mathbb {K}}^{c}}f|^{2}+\sum _{j\in \mathbb {K}}|\Lambda _{j}f|^{2}, \end{aligned}$$

from (17) it follows that

19 $$\begin{aligned}\sum _{j\in \mathbb {J}}|\widetilde{\Lambda }_{j}S_{\mathbb {K}}f|^{2}+\sum _{j\in {\mathbb {K}}^{c}}|\Lambda _{j}f|^{2}&=\sum _{j\in \mathbb {J}}|\widetilde{\Lambda }_{j}S_{{\mathbb {K}}^{c}}f|^{2}+\sum _{j\in \mathbb {K}}|\Lambda _{j}f|^{2}\nonumber \\ &\ge (2\lambda -\lambda ^{2})\langle S_{\mathbb {K}}f,f\rangle +(1-\lambda ^{2})\langle S_{{\mathbb {K}}^{c}}f,f\rangle \nonumber \\ &=(2\lambda -\lambda ^{2})\sum _{j\in \mathbb {K}}|\Lambda _{j}f|^{2}+(1-\lambda ^{2})\sum _{j\in {\mathbb {K}}^{c}}|\Lambda _{j}f|^{2}. \end{aligned}$$

Clearly, $U=S^{-\frac{1}{2}}S_{\mathbb {K}}S^{-\frac{1}{2}}$ and $V=S^{-\frac{1}{2}}S_{{\mathbb {K}}^{c}}S^{-\frac{1}{2}}$ are positive and $UV=VU$. Thus,

$$\begin{aligned} 0\le UV=U(\mathrm{Id}_{\mathscr {H}}-U)=U-U^{2}=S^{-\frac{1}{2}}(S_{\mathbb {K}}-S_{\mathbb {K}}S^{-1}S_{\mathbb {K}})S^{-\frac{1}{2}}, \end{aligned}$$

from which we conclude that $S_{\mathbb {K}}-S_{\mathbb {K}}S^{-1}S_{\mathbb {K}}\ge 0$. Therefore,

$$\begin{aligned}&\sum _{j\in \mathbb {J}}|\widetilde{\Lambda }_{j}S_{{\mathbb {K}}^{c}}f|^{2}+\sum _{j\in \mathbb {K}}|\Lambda _{j}f|^{2}=\sum _{j\in \mathbb {J}}|\widetilde{\Lambda }_{j}S_{\mathbb {K}}f|^{2}+\sum _{j\in {\mathbb {K}}^{c}}|\Lambda _{j}f|^{2}\\&\qquad =\langle S^{-1}S_{\mathbb {K}}f,S_{\mathbb {K}}f\rangle +\langle S_{{\mathbb {K}}^{c}}f,f\rangle \le \langle S_{\mathbb {K}}f,f\rangle +\langle S_{{\mathbb {K}}^{c}}f,f\rangle \\&\qquad =\langle (S_{\mathbb {K}}+S_{{\mathbb {K}}^{c}})f,f\rangle =\langle Sf,f\rangle =\sum _{j\in \mathbb {J}}|\Lambda _{j}f|^{2}. \end{aligned}$$

This completes the proof.

If $\{\Lambda _{j}\in \mathrm{End}^{*}_{\mathcal {A}}({\mathscr {H}},{\mathscr {K}}_{j})\}_{j\in \mathbb {J}}$ is a Parseval g-frame for $\mathscr {H}$ with respect to $\{{\mathscr {K}}_{j}\}_{j\in \mathbb {J}}$, then its g-frame operator *S* is equal to $\mathrm{Id}_{\mathscr {H}}$. For any ${\mathbb {K}}\subset {\mathbb {J}}$ and any $f\in \mathscr {H}$, we have

20 $$\begin{aligned} \sum _{j\in \mathbb {J}}|\widetilde{\Lambda }_{j}S_{\mathbb {K}}f|^{2}&= \sum _{j\in \mathbb {J}}\langle \widetilde{\Lambda }_{j}S_{\mathbb {K}}f,\widetilde{\Lambda }_{j}S_{\mathbb {K}}f\rangle =\sum _{j\in \mathbb {J}}\langle \Lambda _{j}S_{\mathbb {K}}f,\Lambda _{j}S_{\mathbb {K}}f\rangle \nonumber \\&= \sum _{j\in \mathbb {J}}\langle \Lambda ^{*}_{j}\Lambda _{j}S_{\mathbb {K}}f,S_{\mathbb {K}}f\rangle =\langle S_{\mathbb {K}}f,S_{\mathbb {K}}f\rangle =\biggl |\sum _{j\in {\mathbb {K}}}\Lambda ^{*}_{j}\Lambda _{j}f\biggl |^{2}. \end{aligned}$$

Similarly, we have

21 $$\begin{aligned} \sum _{j\in \mathbb {J}}|\widetilde{\Lambda }_{j} S_{{\mathbb {K}}^{c}}f|^{2}=\biggl |\sum _{j\in {\mathbb {K}}^{c}} \Lambda ^{*}_{j}\Lambda _{j}f\biggl |^{2}. \end{aligned}$$

Hence, Theorem 6 leads to a direct consequence as follows.

### **Corollary 7**

*Let *$\{\Lambda _{j}\in \mathrm{End}^{*}_{\mathcal {A}}({\mathscr {H}},{\mathscr {K}}_{j})\}_{j\in \mathbb {J}}$*be a Parseval g-frame for*$\mathscr {H}$*with respect to*$\{{\mathscr {K}}_{j}\}_{j\in \mathbb {J}}$, *then for any*$\lambda \in [0,1]$, *for all*$\mathbb {K}\subset \mathbb {J}$*and all*$f\in \mathscr {H}$, *we have*

22 $$\begin{aligned} \langle f,f\rangle&\ge \biggl |\sum _{j\in \mathbb {K}}\Lambda ^{*}_{j}\Lambda _{j}f\biggl |^{2}+\sum _{j\in {\mathbb {K}}^{c}}|\Lambda _{j}f|^{2}=\biggl |\sum _{j\in {\mathbb {K}}^{c}}\Lambda ^{*}_{j}\Lambda _{j}f\biggl |^{2}+\sum _{j\in \mathbb {K}}|\Lambda _{j}f|^{2}\nonumber \\&\ge (2\lambda -\lambda ^{2})\sum _{j\in \mathbb {K}}|\Lambda _{j}f|^{2}+(1-\lambda ^{2})\sum _{j\in {\mathbb {K}}^{c}}|\Lambda _{j}f|^{2}. \end{aligned}$$

### *Remark 8*

If we take $\lambda =\frac{1}{2}$ in Theorem  6 and Corollary 7, then we can obtain the inequalities in Theorems  1 and  2.

### **Theorem 9**

*Let*$\{\Lambda _{j}\in \mathrm{End}^{*}_{\mathcal {A}}({\mathscr {H}},{\mathscr {K}}_{j})\}_{j\in \mathbb {J}}$*be a g-frame for*$\mathscr {H}$*with respect to*$\{{\mathscr {K}}_{j}\}_{j\in \mathbb {J}}$*with canonical dual g-frame*$\{\widetilde{\Lambda }_{j}\} _{j\in \mathbb {J}}$. *Then for any*$\lambda \in [\frac{1}{2},1]$, *for all*$\mathbb {K}\subset \mathbb {J}$*and all*$f\in \mathscr {H}$, *we have*

23 $$\begin{aligned}&0\le \sum _{j\in \mathbb {K}}|\Lambda _{j}f|^{2}-\sum _{j\in \mathbb {J}}|\widetilde{\Lambda }_{j}S_{\mathbb {K}}f|^{2}\le (2\lambda -1)\sum _{j\in {\mathbb {K}}^{c}}|\Lambda _{j}f|^{2} +(\lambda -1)^{2}\sum _{j\in \mathbb {J}}|\Lambda _{j}f|^{2}. \end{aligned}$$

24 $$\begin{aligned}&(4\lambda -2\lambda ^{2}-1)\sum _{j\in \mathbb {K}}|\Lambda _{j}f|^{2}+(1-2\lambda ^{2})\sum _{j\in {\mathbb {K}}^{c}}|\Lambda _{j}f|^{2}\nonumber \\&\qquad \le \sum _{j\in \mathbb {J}}|\widetilde{\Lambda }_{j}S_{\mathbb {K}}f|^{2}+\sum _{j\in \mathbb {J}}|\widetilde{\Lambda }_{j}S_{{\mathbb {K}}^{c}}f|^{2}\le 2\lambda \sum _{j\in \mathbb {J}}|\Lambda _{j}f|^{2}. \end{aligned}$$

### *Proof*

Let *S* be the g-frame operator of $\{\Lambda _{j}\}_{j\in \mathbb {J}}$. As mentioned before, $S_{\mathbb {K}}-S_{\mathbb {K}}S^{-1}S_{\mathbb {K}}\ge 0$, thus for each $f\in \mathscr {H}$ we have

25 $$\begin{aligned} 0\le \langle S_{\mathbb {K}}f,f\rangle -\langle S^{-1}S_{\mathbb {K}}f,S_{\mathbb {K}}f\rangle =\sum _{j\in \mathbb {K}}|\Lambda _{j}f|^{2}-\sum _{j\in \mathbb {J}}|\widetilde{\Lambda }_{j}S_{\mathbb {K}}f|^{2}. \end{aligned}$$

From (15) it follows that

26 $$\begin{aligned} \sum _{j\in \mathbb {K}}|\Lambda _{j}f|^{2}-\sum _{j\in \mathbb {J}}|\widetilde{\Lambda }_{j}S_{\mathbb {K}}f|^{2}&=\langle S_{\mathbb {K}}f,f\rangle -\langle S^{-1}S_{\mathbb {K}}f,S_{\mathbb {K}}f\rangle \nonumber \\&\le \langle S_{\mathbb {K}}f,f\rangle -2\lambda \langle S_{\mathbb {K}}f,f\rangle +\lambda ^{2}\langle Sf,f\rangle \nonumber \\&=(1-2\lambda )\langle S_{\mathbb {K}}f,f\rangle +\lambda ^{2}\langle Sf,f\rangle \nonumber \\&=(1-2\lambda )(\langle Sf,f\rangle -\langle S_{{\mathbb {K}}^{c}}f,f\rangle )+\lambda ^{2}\langle Sf,f\rangle \nonumber \\&=(2\lambda -1)\langle S_{{\mathbb {K}}^{c}}f,f\rangle +(\lambda -1)^{2}\langle Sf,f\rangle \nonumber \\&=(2\lambda -1)\sum _{j\in {\mathbb {K}}^{c}}|\Lambda _{j}f|^{2}+(\lambda -1)^{2}\sum _{j\in \mathbb {J}}|\Lambda _{j}f|^{2}. \end{aligned}$$

Combination of (25) and (26) yields (23). It remains to prove (24). Using formulas (15) and (17) we obtain

27 $$\begin{aligned}&\sum _{j\in \mathbb {J}}|\widetilde{\Lambda }_{j}S_{\mathbb {K}}f|^{2}+\sum _{j\in \mathbb {J}}|\widetilde{\Lambda }_{j}S_{{\mathbb {K}}^{c}}f|^{2}= \langle S^{-1}S_{\mathbb {K}}f,S_{\mathbb {K}}f\rangle +\langle S^{-1}S_{{\mathbb {K}}^{c}}f,S_{{\mathbb {K}}^{c}}f\rangle \nonumber \\&\qquad \ge 2\lambda \langle S_{\mathbb {K}}f,f\rangle -\lambda ^{2}\langle Sf,f\rangle +(2\lambda -\lambda ^{2}-1)\langle S_{\mathbb {K}}f,f\rangle +(1-\lambda ^{2})\langle S_{{\mathbb {K}}^{c}}f,f\rangle \nonumber \\&\qquad =(4\lambda -2\lambda ^{2}-1)\langle S_{\mathbb {K}}f,f\rangle +(1-2\lambda ^{2})\langle S_{{\mathbb {K}}^{c}}f,f\rangle \nonumber \\&\qquad =(4\lambda -2\lambda ^{2}-1)\sum _{j\in \mathbb {K}}|\Lambda _{j}f|^{2}+(1-2\lambda ^{2})\sum _{j\in {\mathbb {K}}^{c}}|\Lambda _{j}f|^{2}. \end{aligned}$$

Since $S^{-\frac{1}{2}}S_{\mathbb {K}}S^{-\frac{1}{2}}$ and $S^{-\frac{1}{2}}S_{{\mathbb {K}}^{c}}S^{-\frac{1}{2}}$ are positive and self-adjoint, by Lemma 5 we have

28 $$\begin{aligned}&\sum _{j\in \mathbb {J}}|\widetilde{\Lambda }_{j}S_{\mathbb {K}}f|^{2}+\sum _{j\in \mathbb {J}}|\widetilde{\Lambda }_{j}S_{{\mathbb {K}}^{c}}f|^{2}= \langle S^{-1}S_{\mathbb {K}}f,S_{\mathbb {K}}f\rangle +\langle S^{-1}S_{{\mathbb {K}}^{c}}f,S_{{\mathbb {K}}^{c}}f\rangle \nonumber \\&\qquad =\langle S^{-\frac{1}{2}}S_{\mathbb {K}}S^{-\frac{1}{2}}S^{\frac{1}{2}}f,S^{-\frac{1}{2}}S_{\mathbb {K}}S^{-\frac{1}{2}}S^{\frac{1}{2}}f\rangle +\langle S^{-\frac{1}{2}}S_{{\mathbb {K}}^{c}}S^{-\frac{1}{2}}S^{\frac{1}{2}}f,S^{-\frac{1}{2}}S_{{\mathbb {K}}^{c}}S^{-\frac{1}{2}}S^{\frac{1}{2}}f\rangle \nonumber \\&\qquad \le 2\lambda \langle S^{-\frac{1}{2}}S_{\mathbb {K}}S^{-\frac{1}{2}}S^{\frac{1}{2}}f,S^{\frac{1}{2}}f\rangle +2\lambda \langle S^{-\frac{1}{2}}S_{{\mathbb {K}}^{c}}S^{-\frac{1}{2}}S^{\frac{1}{2}}f,S^{\frac{1}{2}}f\rangle \nonumber \\&\qquad =2\lambda \langle S_{\mathbb {K}}f,f\rangle +2\lambda \langle S_{{\mathbb {K}}^{c}}f,f\rangle =2\lambda \langle Sf,f\rangle =2\lambda \sum _{j\in \mathbb {J}}|\Lambda _{j}f|^{2}. \end{aligned}$$

This together with (27) gives (24).

By (20), (21) and above theorem, we immediately get the following result.

### **Corollary 10**

*Let*$\{\Lambda _{j}\in \mathrm{End}^{*}_{\mathcal {A}}({\mathscr {H}},{\mathscr {K}}_{j})\}_{j\in \mathbb {J}}$*be a Parseval g-frame for*$\mathscr {H}$*with respect to*$\{{\mathscr {K}}_{j}\}_{j\in \mathbb {J}}$. *Then for any*$\lambda \in [\frac{1}{2},1]$,*for all*$\mathbb {K}\subset \mathbb {J}$*and all*$f\in \mathscr {H}$, *we have*

29 $$\begin{aligned}&0\le \sum _{j\in \mathbb {K}}|\Lambda _{j}f|^{2}-\biggl |\sum _{j\in \mathbb {K}}\Lambda ^{*}_{j}\Lambda _{j}f\biggl |^{2}\le (2\lambda -1) \sum _{j\in {\mathbb {K}}^{c}}|\Lambda _{j}f|^{2}+(\lambda -1)^{2}\langle f,f\rangle . \end{aligned}$$

30 $$\begin{aligned}&(4\lambda -2\lambda ^{2}-1)\sum _{j\in \mathbb {K}}|\Lambda _{j}f|^{2}+(1-2\lambda ^{2})\sum _{j\in {\mathbb {K}}^{c}}|\Lambda _{j}f|^{2}\nonumber \\&\qquad \quad \le \biggl |\sum _{j\in \mathbb {K}}\Lambda ^{*}_{j}\Lambda _{j}f\biggl |^{2}+\biggl |\sum _{j\in {\mathbb {K}}^{c}}\Lambda ^{*}_{j}\Lambda _{j}f\biggl |^{2}\le 2\lambda \langle f,f\rangle . \end{aligned}$$

### *Remark 11*

The inequalities in Theorems  3 and  4 can be obtained when taking $\lambda =\frac{1}{2}$ in Theorem  9 and Corollary  10.

## Conclusions

In this work, we present several double inequalities with flexible scalars for g-frames in Hilbert $C^{*}$-modules and show that they are more general and cover some existing results.

## References

[CR1] Alijani A (2015). Generalized frames with $$C^{\ast }$$-valued bounds and their operator duals. Filomat.

[CR2] Alijani A, Dehghan MA (2012). G-frames and their duals for Hilbert $$C^{\ast }$$-modules. Bull Iranian Math Soc.

[CR3] Askarizadeh A, Dehghan MA (2013). G-frames as special frames. Turk J Math.

[CR4] Balan R, Casazza PG, Edidin D, Kutyniok G (2007). A new identity for Parseval frames. Proc Am Math Soc.

[CR5] Benedetto JJ, Powell AM, Yilmaz O (2006). Sigma-delta ($$\Sigma \text{-}\Delta $$) quantization and finite frames. IEEE Trans Inform Theory.

[CR6] Candès EJ, Donoho DL (2005). Continuous curvelet transform: II. Discretization and frames. Appl Comput Harmon Anal.

[CR7] Daubechies I, Grossmann A, Meyer Y (1986). Painless nonorthogonal expansions. J Math Phys.

[CR8] Duffin RJ, Schaeffer AC (1952). A class of nonharmonic Fourier series. Trans Am Math Soc.

[CR9] Frank M, Larson DR (2002). Frames in Hilbert $$C^{\ast }$$-modules and $$C^{\ast }$$-algebras. J Oper Theory.

[CR10] Găvruţa P (2006). On some identities and inequalities for frames in Hilbert spaces. J Math Anal Appl.

[CR11] Han DG, Jing W, Larson D, Li PT, Mohapatra RN (2013). Dilation of dual frame pairs in Hilbert $$C^{\ast }$$-modules. Results Math.

[CR12] Jivulescu MA, Găvruţa P (2015). Indices of sharpness for Parseval frames, quantum effects and observables. Sci Bull Politeh Univ Timişoara Trans Math Phys.

[CR13] Khosravi A, Khosravi B (2008). Fusion frames and g-frames in Hilbert $$C^{\ast }$$-modules. Int J Wavelets Multiresolut Inf Process.

[CR14] Poria A (2016) Some identities and inequalities for Hilbert–Schmidt frames. www.arxiv.org, math. FA/1602.07912v1

[CR15] Rashidi-Kouchi M, Nazari A, Amini M (2014). On stability of g-frames and g-Riesz bases in Hilbert $$C^{\ast }$$-modules. Int J Wavelets Multiresolut Inf Process.

[CR16] Sun WC (2006). G-frames and g-Riesz bases. J Math Anal Appl.

[CR17] Sun WC (2010). Asymptotic properties of Gabor frame operators as sampling density tends to infinity. J Funct Anal.

[CR18] Xiang ZQ (2016). New inequalities for g-frames in Hilbert $$C^{\ast }$$-modules. J Math Inequal.

[CR19] Xiang ZQ, Li YM (2016). G-frames for operators in Hilbert $$C^{\ast }$$-modules. Turk J Math.

[CR20] Xiao XC, Zeng XM (2010). Some properties of g-frames in Hilbert $$C^{\ast }$$-modules. J Math Anal Appl.

